# Altered T-Cell Receptor β-Chain and Lactate Dehydrogenase Are Associated With the Immune Pathogenesis of Biliary Atresia

**DOI:** 10.3389/fmed.2021.778500

**Published:** 2021-12-24

**Authors:** Jing Ye, Dengming Lai, Dan Cao, Linhua Tan, Lei Hu, Hua Zha, Jiezuan Yang, Qiang Shu

**Affiliations:** ^1^Department of Surgical ICU, Children's Hospital, National Clinical Research Center for Child Health, Zhejiang University School of Medicine, Hangzhou, China; ^2^Department of Neonatal Surgery, Children's Hospital, National Clinical Research Center for Child Health, Zhejiang University School of Medicine, Hangzhou, China; ^3^State Key Laboratory for Diagnosis and Treatment of Infectious Diseases, National Clinical Research Center for Infectious Diseases, The First Affiliated Hospital, Zhejiang University School of Medicine, Hangzhou, China; ^4^Department of Thoracic and Cardiovascular Surgery, Children's Hospital, National Clinical Research Center for Child Health, Zhejiang University School of Medicine, Hangzhou, China

**Keywords:** biliary atresia, high throughput sequencing, immune repertoire, lactate dehydrogenase, immune pathogenesis

## Abstract

**Background:** Biliary atresia (BA) is considered to be an autoimmune-mediating inflammatory injury. The pathogenesis of BA has been proposed with the clonal transformation of T cells expressing analogous T-cell receptor β-chain variable regions (TRBVs).

**Methods:** The TRBV profile of the peripheral blood mononuclear cells (PBMCs) in infants with BA and control infants (healthy donors, HDs), respectively, were characterized by using high-throughput sequencing (HTS). The diversity of T cells was analyzed based on the frequency of complementarity-determining region 3 (CDR3) or V(CDR3)J. Moreover, the correlation between absolute lymphocyte count (ALC) and lactate dehydrogenase (LDH) or diversity (clonality) indices, respectively, were analyzed for subjects with BA and HD.

**Results:** The diversity indices of CDR3, V(CDR3)J in BA are lower than those in subjects with HD, in addition, there are significantly different levels of neutrophile, neutrophile/lymphocyte ratio (NLR), and LDH between groups of BA and HD. The correlation between ALC and diversity index is significant in subjects with HD but is not for subjects with BA. Conversely, the relationship between ALC and LDH is significant in subjects with BA but is not for subjects with HD. Moreover, 12 CDR3 motifs are deficient or lower expression in BA compared with that in the HD group.

**Conclusion:** Our results demonstrate that the profile of TRBV repertoire is significantly different between subjects with BA and HD, and suggest that the immune imbalance and elevated LDH level are associated with the pathogenesis of BA. Moreover, the values of neutrophile, NLR, and LDH could be used for the differential diagnosis of BA.

## Introduction

Biliary atresia (BA) is characterized by progressive, necroinflammatory, and fibrosis of the extra- and intrahepatic biliary system, leading to the bile flow obstruction, cholestasis, and jaundice in infants (1, 2). The morbidity of BA in Europe and the United States are about 1/18,000 and 1/15,000, respectively, and the incidence in Asia is much higher, such as the incidence in Taiwan China, which is one out of 5,000 live birth (3, 4). BA has a poor prognosis with a high mortality and is the most common indication for the liver transplantation in infants (5). However, the pathogenesis of BA is still vague. Sato K et al. suggested that the biliary epithelial cells (cholangiocytes) were not only a physical barrier that drains the bile into the duodenum but they were also immunocompetent cells involved in tissue homeostasis, which is capable of recognizing microbial conserved antigens through pattern recognition receptors (PRRs) and initiating an inflammatory response (6).

Recent studies suggested that BA may be an immune disease mediated by T cells (7, 8), and T cells and cytokines play a pivotal role in the osteogenesis of BA (9). Through the stimulation of specific antigens, the selective distribution of the T cell receptor (TCR, TR) spectrum could reflect the clonal amplification and functional changes of the T-cell population (10). The complementarity-determining region 3 (CDR3) is the only nongermline coding region of TR, which is the most likely to mutate and the vital position of responding to foreign antigens. The spatial structure of CDR3 is determined by its length and amino acid composition, which is determined by the random combination of variable (V)/diversity (D)/joining (J) gene fragments, and random deletion and/or insertion of nucleotides at the junction of each V/D/J gene fragment. The analysis of the various characteristics of CDR3 can provide key clues for the study of the structure and function related to the different TRs recognizing specific antigens (11, 12).

Lactate dehydrogenase (LDH) mainly exists in the heart, skeletal muscle, and kidney tissues or organs, and the red blood cells (RBCs) also contain relatively rich LDH. LDH can be released into serum when the organs and tissues are destroyed, so that the activity level of LDH in serum can well reflect the biological characteristics of cells with rich LDH, such as proliferation, lesion, and metabolism (13). The increased LDH-A activity was associated with the autoimmune CD8+ T cells in the rheumatoid arthritis (14).

With the development and application of high-throughput sequencing (HTS) and bioinformatics techniques, we profiled of TRBV repertoire of peripheral blood mononuclear cells (PBMCs) from infants with BA and paired healthy donors (HDs), and explored the relationship between the diversity or LDH and absolute lymphocyte count (ALC). These results indicate and further confirm the immune imbalance of BA, and shed new light on the perspective for the diagnosis of BA and new possible targets for the clinical immune treatment of BA.

## Methods And Materials

### Subjects

Between March 2019 and September 2020, 34 infants with BA were enrolled before they were subjected to surgical treatment in the Children's Hospital, Zhejiang University School of Medicine and 38 HDs were enrolled as controls, matched for ethnicity, age, and gender. The BA infants were confirmed by intraoperative cholangiography, expressed as the intrahepatic and extrahepatic bile ducts were not developed, and the gallbladder was small. Moreover, the infantile liver is enlarged, brown, and tough. Simultaneously, all the infants (including HDs) were excluded from cholestasis, other immune diseases, tumors, neonatal hepatitis, and infectious diseases, the more detailed diagnosis and exclusion criteria shown in (15). Additional characteristics of enrolled subjects at the time of the study are shown (Table 1), and the subjects with HD with normal laboratory indicators were enrolled in the same hospital. This project and protocols were approved by the Ethics Committee of the Children's Hospital, Zhejiang University School of Medicine. Informed consent was obtained from the legal guardian of the enrolled infants. Moreover, this study complied with the Declaration of Helsinki (2008).

**Table 1:** Summary of demographic and serum laboratory data of the enrolled subjects.

**Variables**	**BA**	**HD**	***P*-value**
No. subject	34	38	-
Gender (male/female)	17/17	21/17	>0.8134
Age (Days)	57.53 ± 17.83	67.42 ± 23.12	0.1218
TB (μmmol/dl)	152.45 (105.83, 169.38)	11.60 (8.85, 17.83)	<0.0001
DB (μmmol/dl)	73.70 (57.43, 91.15)	2.60 (1.88, 3.93)	<0.0001
ALT (U/L)	108.00(69.25, 208.50)	36.0 (27.75, 48.25)	<0.0001
AST (U/L)	206 (139.25, 267.25)	51 (39.00, 72.25)	<0.0001
GGT (U/L)	429 (245.50, 660.50)	42 (27.00, 56.50)	<0.0001
Albumin (g/L)	36.75 (34.03, 40.65)	40.50 (39.05, 42.70)	0.0014

*The mean ± SD was used to describe the data of the normal distribution, and the data for the skewed distribution was described by the median (25 quantiles and 75 quantiles). The two-tailed independent sample t-test was used for analyzing normal distribution data, and the Mann–Whitney test was used to compare non-normal distribution data. The chi-squared (Fisher's exact) test was used to compare the sex composition between the two groups. BA, biliary atresia; HD, healthy donor; TB, total bilirubin; DB, direct bilirubin; ALT, alanine aminotransferase; AST, aspartate aminotransferase; GGT, gamma-glutamyl transpeptidase*.

### Cytometric, Biochemical, and LDH Assay

The blood cell counts of 34 samples of BA (38 samples of HD) were conducted in a Blood Cell Analyzer (Mindray BC-7500, China), and the biochemical indicators of the liver function were determined in an automatic biochemical analyzer (AU5821, Beckman Coulter, California, USA) in the clinical laboratory center of our unit. The serum concentration of LDH was determined using the lactic acid substrate method according to the reagent instructions of the manufacturer.

### Ribonucleic Acid Isolation and cDNA Synthesis

We randomly selected 11 out of the thirty-four subjects with BA (eight out of 38 subjects of HD) as represents for further analysis of HTS, for the high cost of HTS. The PBMCs were isolated from 2.5 ml peripheral vein blood of each subject using Ficoll density gradient centrifugation. Total RNA was purified from PBMC using Trizol reagent (Invitrogen, Carlsbad, California, USA) complying with the specifications of the manufacturer. The integrity of RNA was determined using the Agilent Bioanalyzer 2100 (Agilent Technologies, California, USA). Concentrations of RNA were determined using an Eppendorf BioPhotometer Plus (Eppendorf, Hamburg, Germany), and 100 ng of total RNA of each sample was reverse transcription into complementary DNA (cDNA) using the RevertAid First Strand cDNA Synthesis Kit (MBI, LTU).

### Construction of Sequencing Library

Preparation of TRBV CDR3 sequencing library, two-round nested amplicon arm polymerase chain reaction (PCR) was carried out with a Multiplex PCR Assay Kit Ver. 2 (TaKaRa, Dalian, China), the specific primers against each variable and constant gene both were used. For the detailed operation steps, please refer to (16).

### T-cell Receptor β-chain Variable Regions Repertoire Sequencing

Polymerase chain reaction products were further purified by agarose gel electrophoresis according to the specification of the manufacturer, the purified PCR products were sequenced on the Illumina HiSeq × 10 platform (Illumina, San Diego, CA, USA) using specialized sequencing primers with various sample barcodes, and with a read length of 2 × 150 bps.

### Sequencing Data Analysis

The primary data obtained from the Illumina HiSeq × 10 platform was changed to raw paired-end sequence reads by filtering out the low-quality sequences. V, J, and CDR3 fragments of TRBV common sequences were identified using BLAST (+) in the international ImMunoGeneTics (IMGT) (http://www.imgt.org/) by an authorized algorithm (17).

### Diversity Indices Analysis

The diversity of TRBV was evaluated by the Shannon entropy (SE) (diversity) and Simpson index, which have been widely used for evaluating the richness and diversity of TR as described (18). As shown in the formula later, the SE considers account both the number of T-cell clonotype “*n*” and the frequency “*pi*” of each clonotype, where “*pi*” is the frequency of the *i*-th clone in the TR repertoire with *n* clones. SE can determine the diversity of T cells as it reflects the CDR variability. The higher the index, the higher the diversity of T cells in the involved sample.

Shannon entropy (SE):


$$\begin{array}{l} SE=-\overset{n}{\underset{i\;=\;1}{\sum }}Pi\ln\;Pi \end{array}$$

The D50 could be described as that semiquantitative amplified immune repertoire (IR) and sequencing results are sorted and arranged according to the highest to the lowest expression frequency, then added from high to a low level, when the result of addition to 50% (reads), observe how many different CDR3 sequences (clones) are included. The percentage of the number of clones in the total clones is the D50 value.

To monitor the TCR repertoire similarity, exploiting a quantitative index, named Baroni-Urbani and Buser (BUB) index (19, 20), which was determined according to respective formulae (20), and the detailed method was described previously (20, 21). Moreover, the high-expanded clone (HEC) frequency is also used for the description of the IR. The HEC ratio is determined as the total of the abundance of all sequences with a higher abundance than the threshold value. The threshold usually is 0.01 or 0.1%, and its threshold value can be adjusted to comply with the need of the study (22).

High-expanded clone (HEC):


$$\begin{array}{l} HEC=\overset{n}{\underset{i\;=\;1}{\sum }} \end{array}$$

### Statistical Analysis

Indices of normal distribution were described by the mean ± SD and indices of nonnormally distribution were described by the mean ± SEM (standard error of mean) or the Box and whiskers (25 quantiles and 75 quantiles). Differences between the groups were compared using the Mann–Whitney *U* tests or independent sample *t*-tests with two-tailed. The chi-squared test was used to compare the number of TRBV deletions between groups. Logistic analysis was used to analyze the relationship between the specific clone expression (blood cell count) level and the status of BA. The area under the receiver operating characteristic (ROC) curve was used to identify the signature diagnosis index. Correlations between variables were analyzed using the Spearman's rank test. All the statistical analyses were calculated using the GraphPad Prism version 8.0 (GraphPad Software, California, USA) and the SPSS version 20.0 (IBM Corporation, New York, USA). *p* < 0.05 was considered as statistically significant.

## Results

### Sample Amplification and Diversity

**Figure 1:**
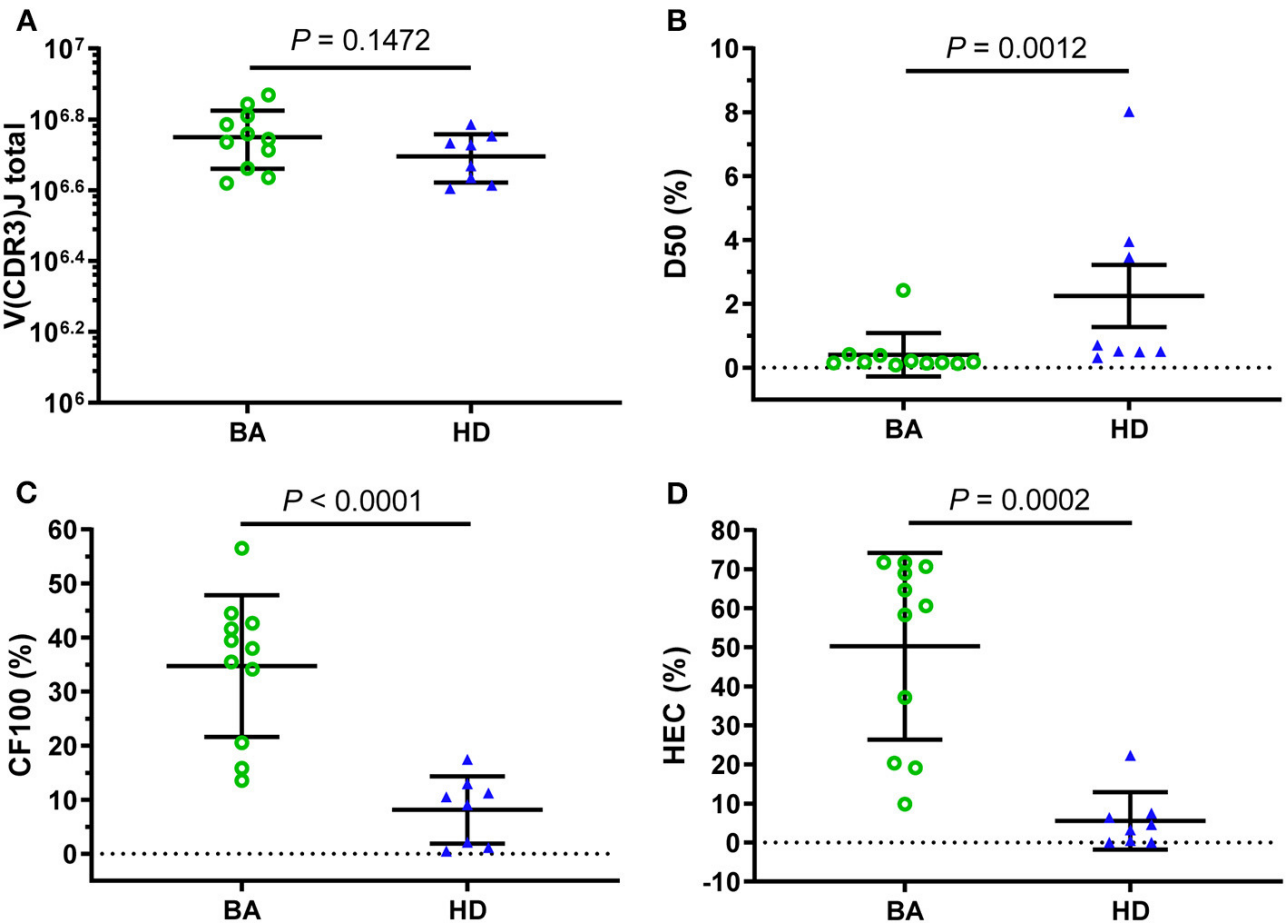
Clonal amplification of T-cell receptor β-chain variable regions (TRBVs) repertoires for Biliary atresia (BA) and healthy donors (HDs). Based on the total V(CDR3)J combinations **(A)**, the clonal amplification of TRBV diversity of each group was described by the D50 **(B)**, CF100 **(C)**, and high-expanded clone (HEC) **(D)**. The bars depict the mean (± SEM or SD) of the groups. D50, the ratio between the numbers of unique CDR3 that make up 50% of the total reads and the total number of unique CDR3 reads; CF100 (cumulative frequency top 100), the cumulative percentage of the most 100 frequents; HEC, the sum of the abundance of all the sequences with the abundance higher than the threshold (0.01 or 0.1%).

In total, approximately 5,622,653 (mean) total V(CDR3)J combinations were generated in BA samples, which was higher than that in HDs (4,954,891, mean), but the difference was no significant difference (*p* = 0.1472, Figure 1A). In addition, the number of unique V(CDR3)J combinations of HDs was significantly higher than that of BA (Supplementary Figure S1). Furthermore, to produce comprehensive unrestricted profiles of V(CDR3)J repertoire diversity, D50 (Figure 1B), CF100 (Cumulative frequency top 100, Figure 1C), and HEC (Figure 1D) were compared between the BA and HDs groups based on the V(CDR3)J frequency and demonstrated significant differences for these indices.

**Figure 2:**
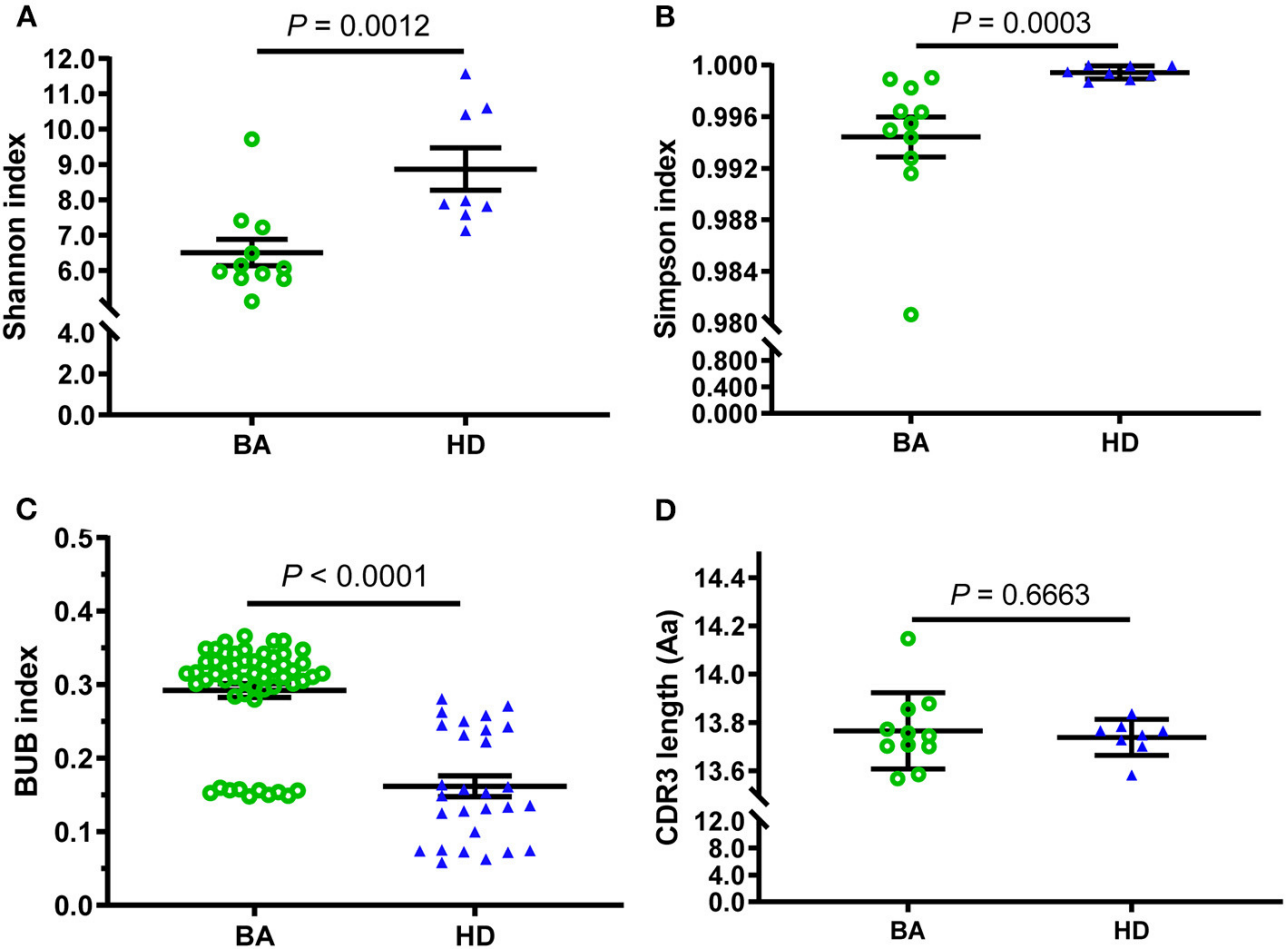
Comparison of diversity and consistency of CDR3 repertoires between BA and HD. The diversity of each group was measured by Shannon index **(A)**, Simpson index **(B)**, the consistency of each group measured by Baroni-Urbani and Buser (BUB) index **(C)**, and comparison of CDR3 average length distribution **(D)**. The bars depict the mean (± SEM or SD) of the groups.

In addition, the Shannon and Simpson indices were also used to evaluate the diversity of CDR3 repertoire and the two indices of BA were significantly lower than those in HDs group (Figures 2A,B). Furthermore, the BUB index was used to determine concordance between each TRBV repertoire of subjects, and the BUB index within subjects with BA was significantly higher than that of HDs (Figure 2C). Moreover, the Jaccard similarity index based on CDR3 or V(CDR3)J was used to see the amino acid sequence similarity among subjects with BA and HDs, which demonstrated higher index in subjects with BA than that in HDs (Supplementary Figure S2)). In addition, the range of CDR3 AA length is almost normal distribution (Gaussian distribution) in both the BA and HDs groups and there is no significant difference between them (Figure 2D).

### Receiver Operating Characteristic Curve Construction

**Figure 3:**
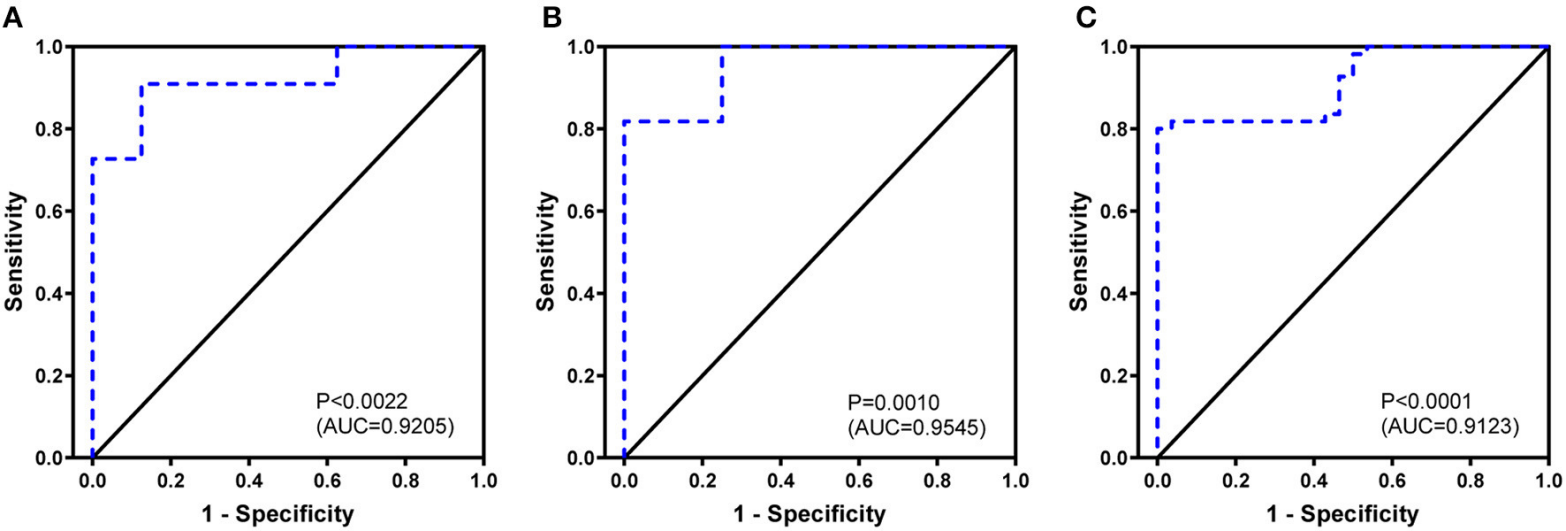
Biliary atresia discriminating receiver operating characteristic curve using the Shannon index **(A)**, Simpson index **(B)**, and BUB index **(C)** based on complementary-determining region 3.

To the ROC curve, a set of diversity indexes (Shannon index, Simpson index, and BUB index) showed efficient performance in identifying cases with BA. The leave-one-out cross-validation yielded an area under the curve (AUC) of Shannon index is 0.9205 (95% CI: 0.7936–1.0000) (Figure 3A); Simpson index is 0.9545 (95% CI: 0.8683–1.0000) (Figure 3B), and BUB index is 0.9123 (95% CI: 0.8527–0.9720) (Figure 3C). The distinction between BA and HDs groups promised the possibility of developing these indexes for an early diagnosis of BA. Furthermore, the cutoff value is expressed as, Shannon index, 0.7501; Simpson index, 0.9984; and BUB index, 0.2822, respectively.

### Correlation Between the TR Repertoire and Clinical Indicators

**Figure 4:**
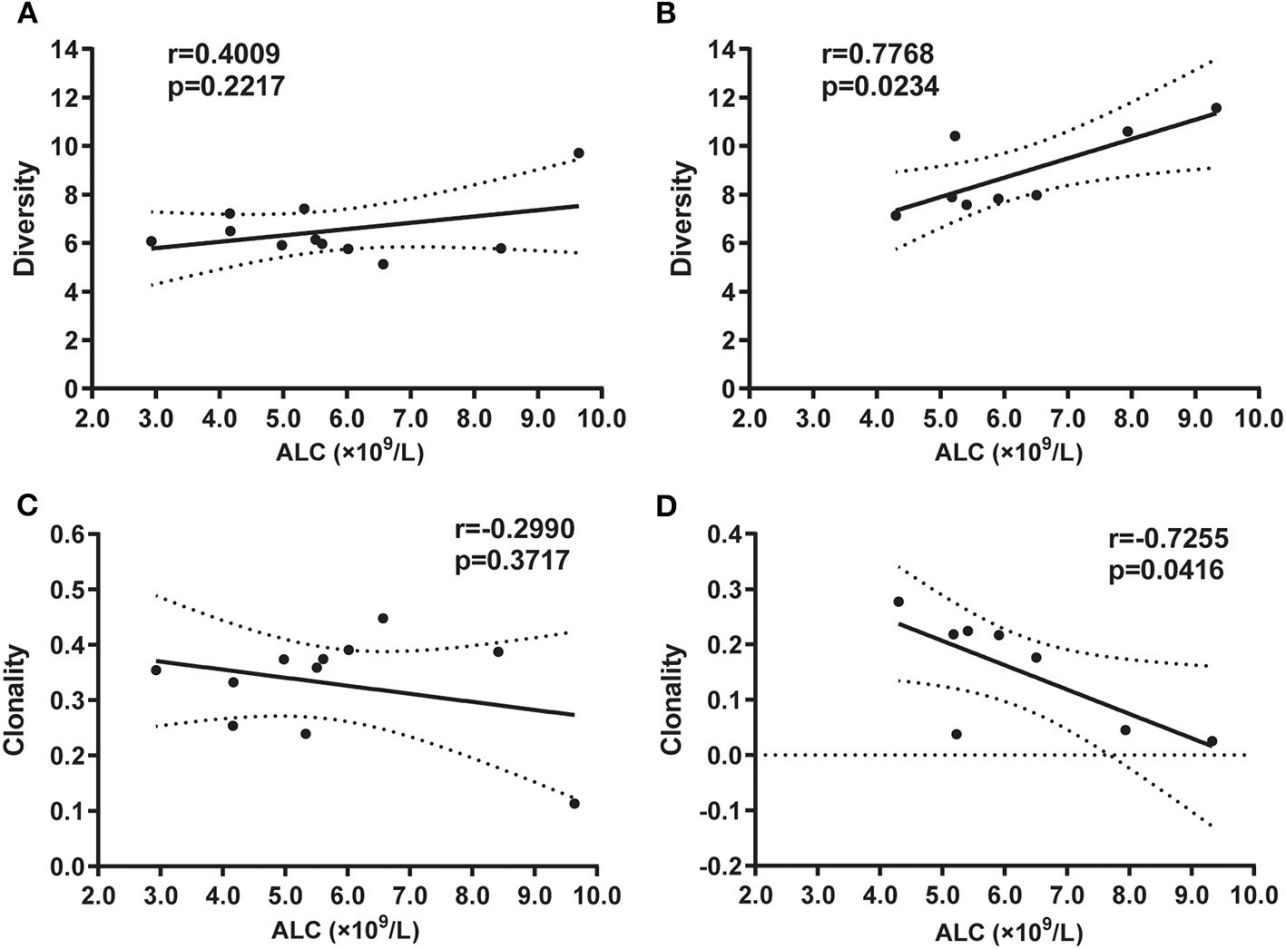
Correlation between absolute lymphocyte count (ALC) and diversity in BA **(A)** or in HD **(B)** groups, and clonality in BA **(C)** or HD **(D)** groups, respectively. Statistical analysis was performed using the Spearman's rank test. The diversity index usually refers to SE that can measure the diversity of T-cell clones as it reflects the CDR variability. The higher the index, the higher the diversity of T-cell clones in the sample. Clonality is based on the normalized SE, which is inversely related to the diversity of T-cell clones. The clonality value is always between 0 and 1.

To further investigate how peripheral lymphocyte diversity reflects immune status in BA, we evaluated the relationship between ALC and CDR3 diversity (or clonality) and demonstrated that the positive correlation between ALC and diversity index was no significant for the BA group (Figure 4A), but for HDs group, the positive correlation was significant (Figure 4B). Similarly, the negative correlation between ALC and clonality was of no significance in the BA group (Figure 4C), but for HDs group, the negative correlation was significant (Figure 4D).

**Figure 5:**
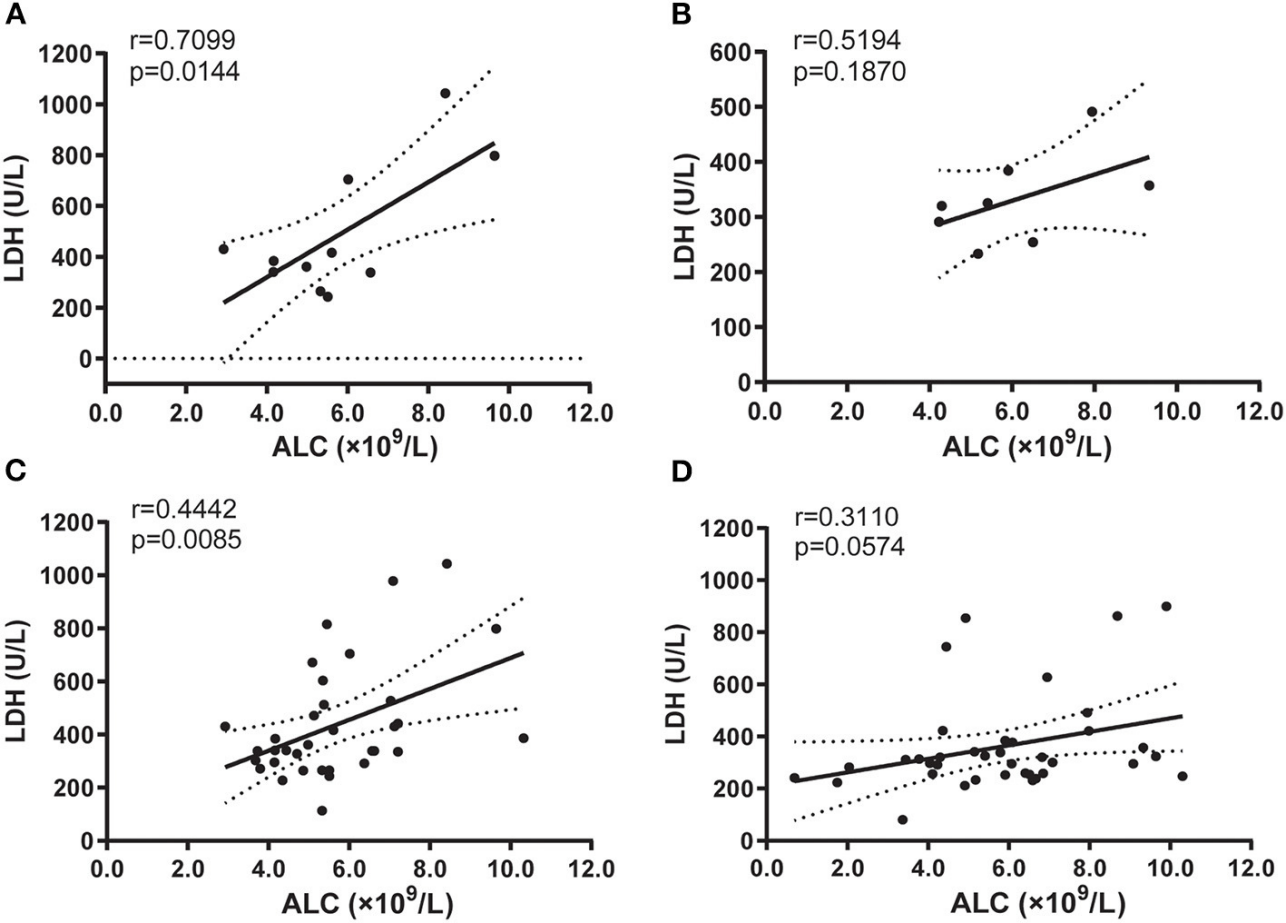
Correlation between ALC and lactate dehydrogenase (LDH) in BA **(A)** or in HD **(B)** groups, and groups with expanded cases in BA **(C)**, or in HD **(D)**, respectively. Statistical analysis was performed using the Spearman's rank test.

We then evaluated the two systemic inflammation biomarkers, LDH, neutrophil/lymphocyte ratio (NLR), which represents a worsened prognosis and immune status at high levels (23). We found a significant positive correlation between ALC and LDH in the BA group both with small and expanded cases (Figures 5A,C), but not in the HDs group (Figures 5B,D). However, there are no significant correlations between clonotype or diversity and LDH in both BA and HDs groups (Supplementary Figure S3), similarly, there are no significant correlations between clonotype or diversity and NLR (Supplementary Figure S4). In addition, there was no significant different level of ALC between BA and HDs groups (Supplementary Figure S5A), the levels of neutrophile, NLR, and LDH were significantly higher in BA than those in the HDs groups (Supplementary Figures S5B–D).

### Clonotype Counts at the Different Distribution Frequency of V(CDR3)J

**Figure 6:**
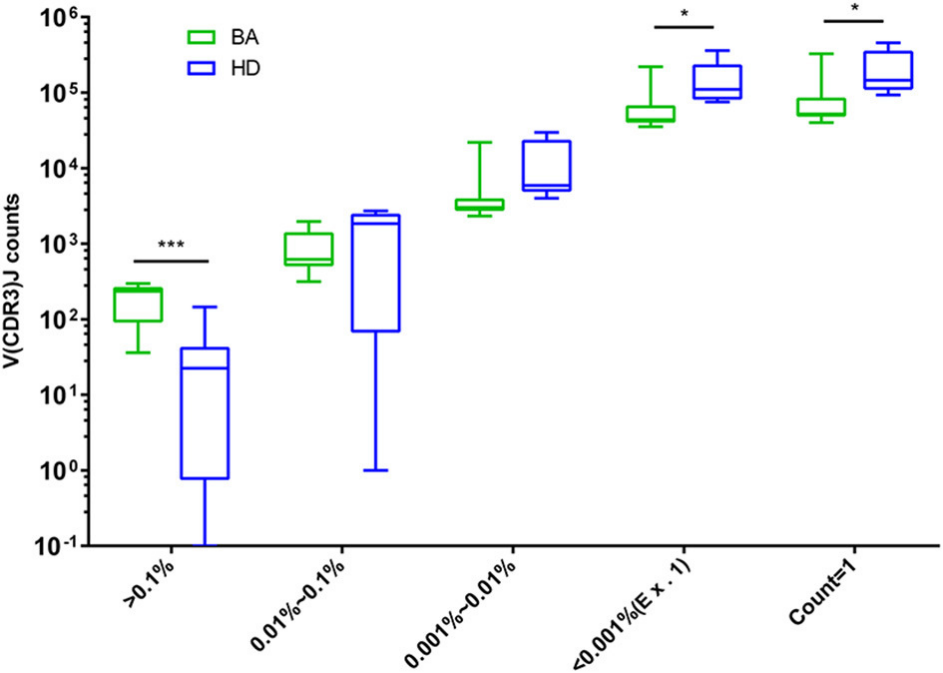
Comparison of the number of V(CDR3)J with different distribution frequency. The number of V(CDR3)J combinations in different distribution frequency is expressed as Box and whiskers (5–95% percentile), in which the middle solid line is the median count of V(CDR3)J, the highest and lowest horizontal lines represent the 5th and 95th percentiles. The *p*-values of comparisons were calculated using the Mann–Whitney nonparametric test, ***p < 0.001; *p < 0.05.

The abundant and diversity of different TRBV clonotypes were presented by V(CDR3)J. The number of V(CDR3)J nucleotide (nt) sequences were divided into five groups based on their frequency distribution (> 0.1%, 0.01–0.1%, 0.001–0.01%, <0.001% (Ex. 1), and count = 1). The results showed that high-abundance clones (i.e., frequency > 0.1%) of BA were higher abundant than that of the HDs group, while low-abundance clones (i.e., frequency < 0.001% and count = 1) were less frequent in individuals with BA (Figure 6), suggesting putatively decreased TRBV diversity in BA group.

### Asymmetric Expression of V/D/J Gene Combinations

We then evaluated the V/D/J gene segment combinations and distribution in subjects with BA and HDs. Some V(D)J combinations presented asymmetric distribution between the two groups, three V(D)J combinations were only found in the BA group, but not in the HDs group, conversely, three V(D)J combinations were only found in the HDs group, but not in the BA group (Table 2). Moreover, the usage of V and VJ combinations demonstrated significantly high levels in HDs compared with those in subjects with BA (Supplementary Figure S6).

**Table 2:** V/D/J gene combinations with asymmetric expression.

**V**	**D**	**J**	**Normalized expression in BA**	**Normalized expression in HD**	**No. of HD samples**	**No. of BA samples**
TRBV14	TRBD2	TRBJ1-6	0.19 ± 0.338	-	0	3
TRBV26	TRBD1	TRBJ2-3	0.161 ± 0.216	-	0	4
TRBV14	TRBD2	TRBJ1-4	0.197 ± 0.353	-	0	3
TRBV5-3	TRBD2	TRBJ2-5	-	0.554 ± 0.832	3	0
TRBV3-1	TRBD1	TRBJ2-3	-	0.285 ± 0.424	3	0
TRBV26	TRBD2	TRBJ2-4	-	0.199 ± 0.263	3	0

*The V(D) combinations listed above are widely expressed (n ≥ 3) in one group while not expressed at all in the other group. The expression frequency is RPM (reads per million). BA, biliary atresia; D, diversity; HD, healthy donor; J, joining; V, variable*.

### Characteristic Motif in TRBV V(CDR3)J

There are 23 V(CDR3)J combinations with more expression in HDs compared with the BA group, which means that a special V(CDR3)J combination is detected in all or the most individuals with HDs, but not in individuals with BA. In addition, 12 out of 23 V(CDR3)J combinations are significantly low expression in the group with BA. Moreover, multivariate Logistic analyses indicated that the 12 CDR3 motifs (CASSSSYEQYF, CASSLGDTQYF, CSDLGWEHLVQVLYNCESGALSKESF, CASSLTDTQYF, CASSPSYEQYF, CASSLGGYEQYF, CASSLQNTEAF, CAGSLGETQYF, CASSLGEAQYF, CASSYSGSSYNEQF, CASSLLANTGELF, and CASSLGETQF) be protective factors for BA genesis (Table 3). Furthermore, the GLIPH2 software (http://50.255.35.37:8080/project) was used to cluster the TRBV sequences of subjects with BA and HDs (24), and the results indicated that subjects with BA and HDs be clustered based on V(CDR3)J frequency, which could separate BA from HDs (Supplementary Table S1).

**Table 3:** Characteristic motifs in TRBV V(CDR3)J of infants of BA.

**No**.	**V**	**CDR3**	**J**	**Freq. of HD**	**Freq. of BA**	**OR**	**95% CI**	***P*-value**	**log_**2**_FC^**a**^**	***p*-value (*t*-test)^**b**^**
1	TRBV28*01	CASSSSYEQYF	TRBJ2-7*01	8/8	5/11	0	0–0.5886	0.0181	−2.833	0.1581
2	TRBV20-1*01	CASDLGWEHLVQVLYNCESGALSKESF	TRBJ2-3*01	8/8	11/11	NA	NA	>0.9999	−0.029	0.2149
3	TRBV12-3*01	CASDLGWEHLVQVLQDRELGSTAKKQF	TRBJ1-4*01	8/8	6/11	0	0–0.8801	0.0445	0.065	0.4124
4	TRBV12-3*01	CASSLGDTQYF	TRBJ2-3*01	8/8	4/11	0	0–0.4111	0.0128	−0.161	0.6096
5	TRBV7-9*02	CASSDLGWEHVFQVLQYGQPRAF	TRBJ2-2*01	8/8	10/11	0	0–12.38	>0.9999	0.423	0.9495
6	TRBV6-5*01	CASDLGWEHVFQVLQYGQPRAF	TRBJ2-2*01	8/8	11/11	NA	NA	>0.9999	0.177	0.5749
7	TRBV29-1*01	CSDLGWEHLVQVLYNCESGALSKESF	TRBJ1-1*01	8/8	4/11	0	0–0.4111	0.0128	−0.909	0.1310
8	TRBV28*01	CASSLGYEQYF	TRBJ2-7*01	8/8	9/11	0	0–2.917	0.4854	3.246	0.3141
9	TRBV5-1*01	CASSDLGWEHLVQVLYNCESGALSKESF	TRBJ1-1*01	8/8	9/11	0	0–2.917	0.4854	0.019	0.4887
10	TRBV29-1*03	CSDLGWEHVFQVLQYGQPRAF	TRBJ2-2*01	8/8	7/11	0	0–1.474	0.1032	−0.477	0.1760
11	TRBV7-9*01	CASSLGGNTEAF	TRBJ1-1*01	8/8	10/11	0	0–12.38	>0.9999	2.551	0.4396
12	TRBV28*01	CASSLTDTQYF	TRBJ2-3*01	8/8	4/11	0	0–0.4111	0.0128	−2.881	0.2769
13	TRBV28*01	CASSPSYEQYF	TRBJ2-7*01	8/8	5/11	0	0–0.5886	0.0181	−3.970	0.1224
14	TRBV28*01	CASSSYNEQF	TRBJ2-1*01	8/8	9/11	0	0–2.917	0.4854	3.761	0.3243
15	TRBV7-9*02	CASSLGGYEQYF	TRBJ2-7*01	8/8	3/11	0	0–0.2907	0.0034	−3.495	0.2820
16	TRBV7-9*02	CASSLQNTEAF	TRBJ1-1*01	8/8	3/11	0	0–0.2907	0.0034	−5.263	0.1432
17	TRBV28*01	CAGSLGETQYF	TRBJ2-5*01	5/8	0/11	0	0–0.3816	0.0048	NA	0.0245
18	TRBV28*01	CASSLGEAQYF	TRBJ2-5*01	4/8	0/11	0	0–0.6787	0.0181	NA	0.0364
19	TRBV6-5*01	CASSYSGSSYNEQF	TRBJ2-1*01	4/8	0/11	0	0–0.6787	0.0181	NA	0.0380
20	TRBV7-4*01	CASSLLANTGELF	TRBJ2-2*01	5/8	1/11	0.06	0.0048–0.6389	0.0408	−5.003	0.0435
21	TRBV7-2*04	CASSFSYEQYF	TRBJ2-7*01	7/8	5/11	0.119	0.0093–1.047	0.1473	−5.239	0.0467
22	TRBV5-1*01	CASSLGETQYF	TRBJ2-5*01	8/8	7/11	0	0–1.474	0.1032	−2.629	0.0468
23	TRBV28*01	CASSLGETQF	TRBJ2-3*01	4/8	0/11	0	0–0.6787	0.0181	NA	0.0470

*a, Logarithm of the ratio of BA to HD (base 2); b, p value for the comparison of the expression between BA and HD; the P-value (<0.05) is underlined*.

*CDR3, complementarity-determining region 3; TRBV, T-cell receptor β-chain variable region*.

## Discussion

Adaptive immunity is an immune response produced by the activation of antigen-specific T/B cells into effector cells and the secretion of cytokines. Numerous studies have suggested that the specific immune response plays a vital role in the mechanism of persistent progression of the injury of liver in BA (25). In this study, we have profiled the TRBV repertoire of BA and revealed disease-related T cells [determined by V(CDR3)J] and provided several potential targets for immunotherapy of BA. The specific lower expressed CDR3b motif may be associated with the BA status, and the higher LDH would be involved in the pathogenesis of BA. Moreover, the Shannon, Simpson, and BUB indices could be used for differential diagnosis of BA from HDs.

T-cell-mediated immune response may be closely related to the pathogenesis of BA. TR is the functional unit of T-cell immune recognizing and responding to the foreign antigen presented by MHC (26, 27). By measuring the diversity and abundance of a specific CDR3 sequence, we can acquire the following results, such as the frequency changes in the different stages of a disease, and the degree of clonal expansion of corresponding T cells, to know the T-cell function and composition of a host (28). In this study, the results with the comparison of D50, CF100, and HEC between BA and HDs indicated that V(CDR3)J be less diversity in BA groups compared with that of HDs, which was confirmed by the Shannon index, Simpson index. Moreover, BUB index is a concordance index, which ranges from 0 to 1, with 0 denoting completely separate populations and one denoting two populations in complete concordance. The higher value of the BUB index in the BA group indicates lower diversity of BA. The lower diversity suggested that the variety of T clonotypes be impaired in subjects with BA. The T clonotypes were from the abnormal clonal amplification of T-cell population, and may be resulted from the host immune responding to foreign virus antigen and self-occult antigens (from bile duct epithelial cells) (29).

Intraoperative cholangiography and liver biopsy are the gold standard for the diagnosis of BA, and a definitive diagnosis of BA can be made while the cholangiography fails to show the biliary tract. But this way has the disadvantages including invasion, complex operation and many complications, which is also not conducive to the early diagnosis of BA. Moreover, the ultrasonographic results of abnormal gall bladder and triangular cord sign have been widely used to identify BA early, but the accuracy of these measurements varies (30). Our results, the significant difference of parameters (Shannon, Simpon, and BUB index) between BA and HDs groups presented a differential diagnostic value for BA, which was also confirmed by their ROC curve analysis. Moreover, the outline of Upset plot based on the number of CDR3 counts presented obvious difference between subjects with BA and HDs (Additional file 2) (31).

A recent study reported that high LDH and NLR correlated with poor outcomes after checkpoint inhibitor treatment in lung cancer (23). In addition, serum LDH level is a cost-effective prognostic biomarker in the patients with operable biliary tract cancers (BTCs) (32), and may predict clinical outcome in patients with BTC receiving first-line chemotherapy (33). In this study, we report that high LDH level is closely related to BA status for the first time, which is partly consistent with the high LDH level of patients with BTC and the reason may be that both BA and BTC be associated with inflammatory process (25). Moreover, the LDH can inhibit CD8+ T-cell antitumor immunity, which is attributed to LDH-modulating cytokine-mediated T-cell differentiation (34). In addition, the diversity of CDR3b was decreased or even missed in BA compared with HDs, which would be related to the increased level of LDH in subjects with BA, and suggested that LDH be involved in the pathogenesis of BA, although, this needs more cases to verify this hypothesis. Moreover, the positive relationship between ALC and LDH is significant in BA, but no significance in HDs, which further verified that LDH plays an important role in the pathogenesis of BA.

In addition, it indicated that the neutrophile being associated with the disease progression of BA that the elevated levels of neutrophile, NLR, and no significant change of lymphocyte counts in subjects with BA. Meng XY et al. reported that the subgroup analysis indicated that for infants with an elevated NLR, the recipient survival was significantly lower when their age > 6 months or PELD > 20 (35). Their results indicate that infants with higher baseline NLR value have lower survival rate of 3 years after transplantation. Further investigations about broaden the application of pre- and post-transplant NLR to guide nutrition intervention and immunosuppression therapy are necessary.

It has also been reported that the efficacy treatment of BA depends on suitable immune phenotypes (36), and the immune balance of organism is vital in maintaining the healthy status of the body (37). The diversity of T cells are presented by the diversity of CDR3b, the more T cells corresponding with more diversity of TR, in this study, which is presented by the significant positive relationship between ALC and diversity of CDR3b in subjects with HDs, however, the relationship was lesioned in subjects with BA. These results indicate the immune imbalance be contributed to the pathogenesis of BA.

Furthermore, 12 TRBV CDR3s are widely shared in subject with HDs, but are seldom or missed in some individuals with BA. These results suggest that the 12 CDR3s be key role in avoiding immune pathogenesis of BA, and provide important molecular targets and theoretical basis for BA immunotherapy. If the results could be confirmed in the expanded cases, which will be possible to delay or treat the immune injury process of BA and improve the clinical outcome by specific T cells produced through specific TR gene transfection.

## Conclusion

In summary, although there are some limitations including limited number of cases in this study, if these findings could be confirmed in a larger range of *in vivo* and *in vitro* tests, if we can further explore the features of these TRs containing specific CDR3 sequences and the correlation between LDH and BA status. Undoubtedly, it would provide an important molecular target for BA immunotherapy, and contribute to further clarifying the immune pathogenesis of BA, and differential diagnosis of BA.

## Data Availability Statement

The datasets for this study can be found in the National Center for Biotechnology Information 300 (NCBI) Human sample from Homo sapiens-BioSample-NCBI (nih.gov). Available at: https://www.ncbi.nlm.nih.gov/biosample/?LinkName=bioproject_biosample_all&from_uid=718110.

## Ethics Statement

The studies involving human participants were reviewed and approved by the Ethics Committee of the Children's Hospital, Zhejiang University School of Medicine. Written informed consent to participate in this study was provided by the participants' legal guardian/next of kin. Written informed consent was obtained from the individual(s), and minor(s)' legal guardian/next of kin, for the publication of any potentially identifiable images or data included in this article.

## Author Contributions

JYe, DL, JYa, and QS gave the study concept and design. JYe, DL, DC, LT, LH, HZ, JYa, and QS were involved in the data acquisition and analysis. DL, DC, LT, LH, and HZ drafted the manuscript and drew the figures. All the authors carefully reviewed and approved the submitting manuscript.

## Funding

This study was supported by Zhejiang Provincial Natural Science Foundation of China (Grant No. LY19H190004).

## Conflict of Interest

The authors declare that the research was conducted in the absence of any commercial or financial relationships that could be construed as a potential conflict of interest.

## Publisher's Note

All claims expressed in this article are solely those of the authors and do not necessarily represent those of their affiliated organizations, or those of the publisher, the editors and the reviewers. Any product that may be evaluated in this article, or claim that may be made by its manufacturer, is not guaranteed or endorsed by the publisher.
