# Supplementary material for: Altered T-Cell Receptor β-Chain and Lactate Dehydrogenase Are Associated With the Immune Pathogenesis of Biliary Atresia
Source: Front Med (Lausanne). 2021 Dec 24;8:778500. doi: 10.3389/fmed.2021.778500 (PMC8739481; doi:10.3389/fmed.2021.778500)
Supplement: Supplementary file 3 [file Data_Sheet_1.docx]

Supplementary Files

Supplementary Figures


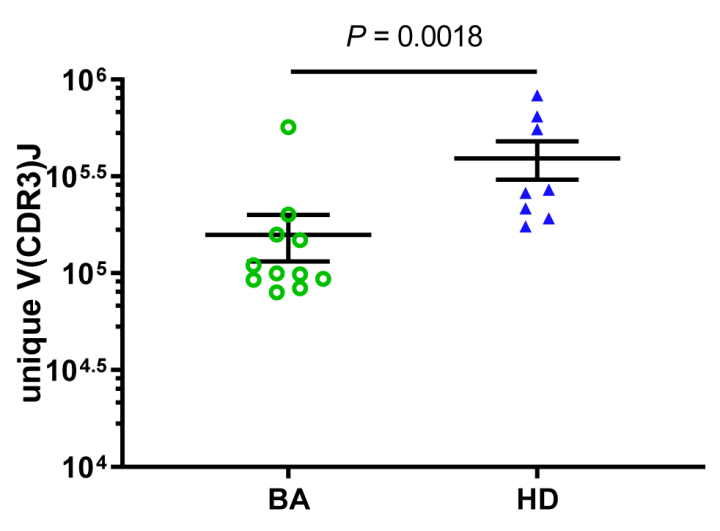


## Fig. S1 Comparison of the unique V(CDR3)J combinations between BA and HD


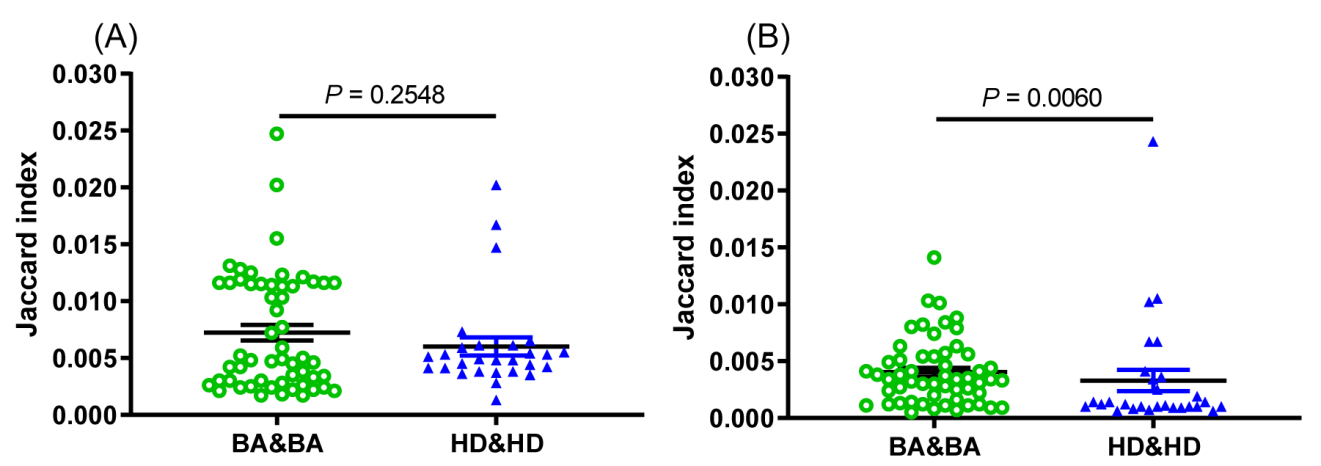


## Fig. S2 Comparison of Jaccard similarity index between BA and HD

The Jaccard similarity index was calculated based on the sequences of CDR3 (A) and V(CDR3)J (B), respectively.


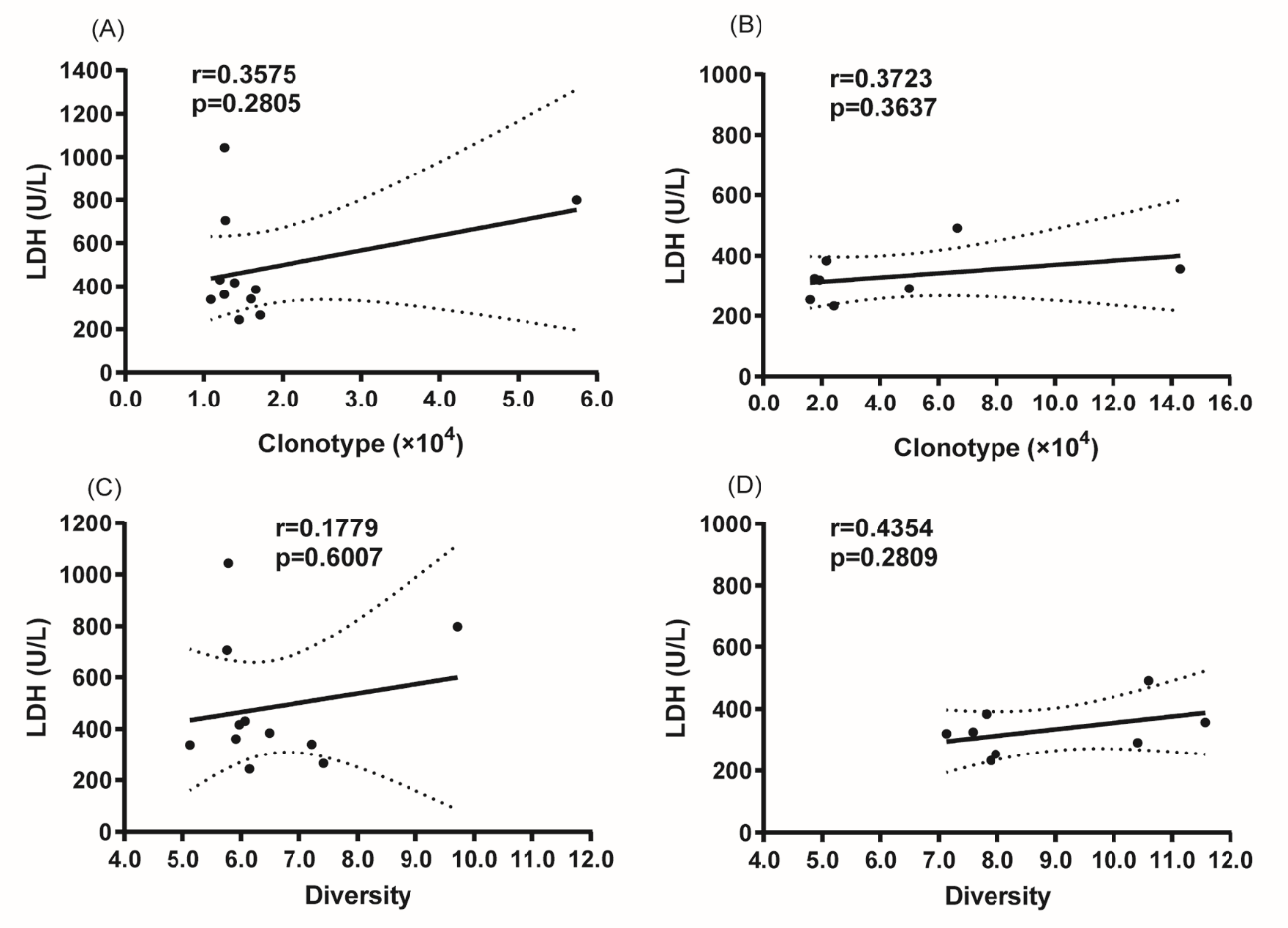


## Fig. S3 Correlation between clonotype and LDH in BA (A) or in HD (B) groups, between diversity and LDH in BA (C) or in HD (D) groups, respectively. Statistical analysis was performed using Spearman’s rank test.


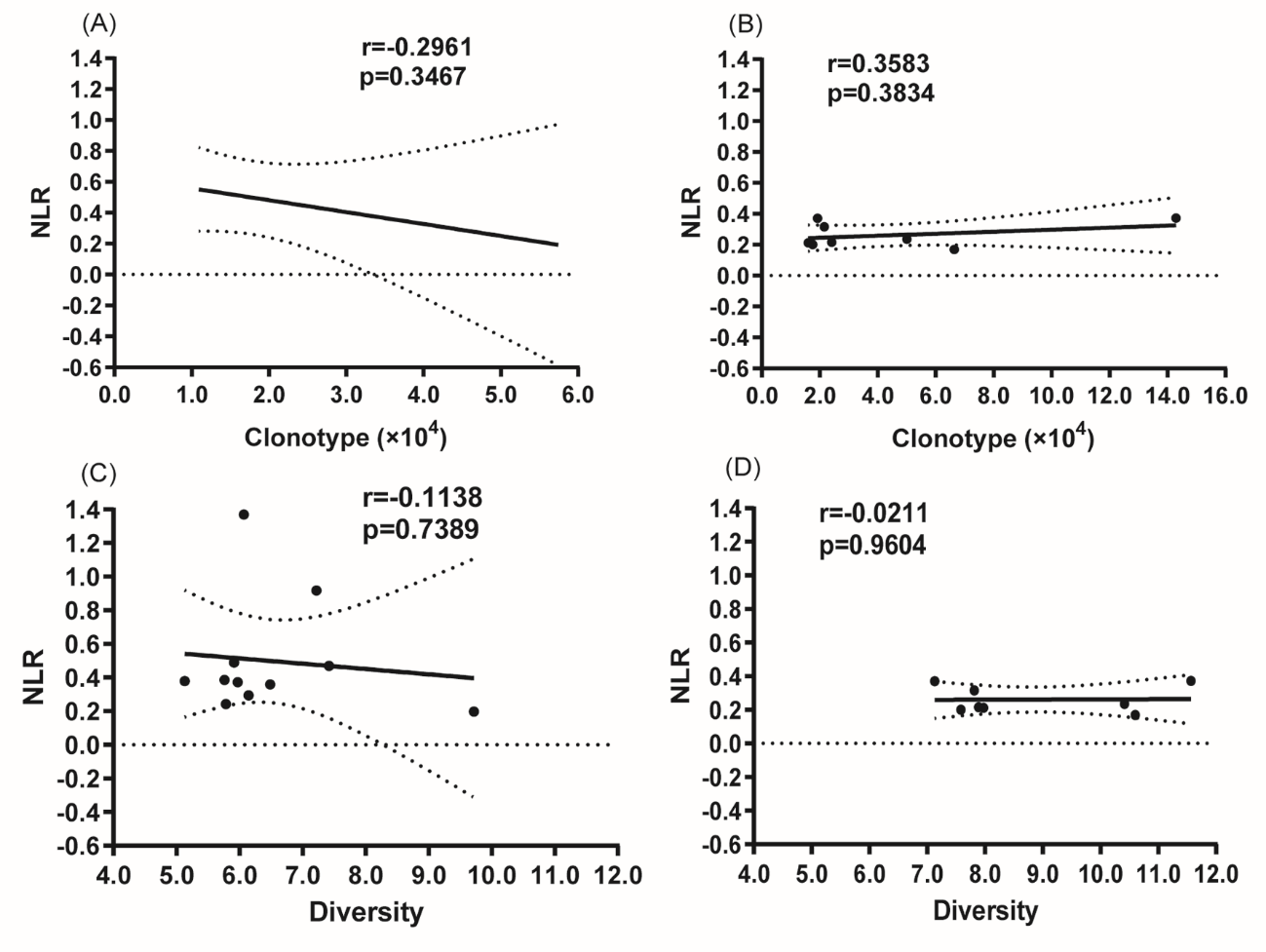


## Fig. S4 Correlation between clonotype and NLR in BA (A) or in HD (B) groups, between diversity and NLR in BA (C) on in HD (D) groups, respectively.

NLR means the ratio of the number between neutrophiles and lymphocytes. Statistical analysis was performed using Spearman’s rank test.


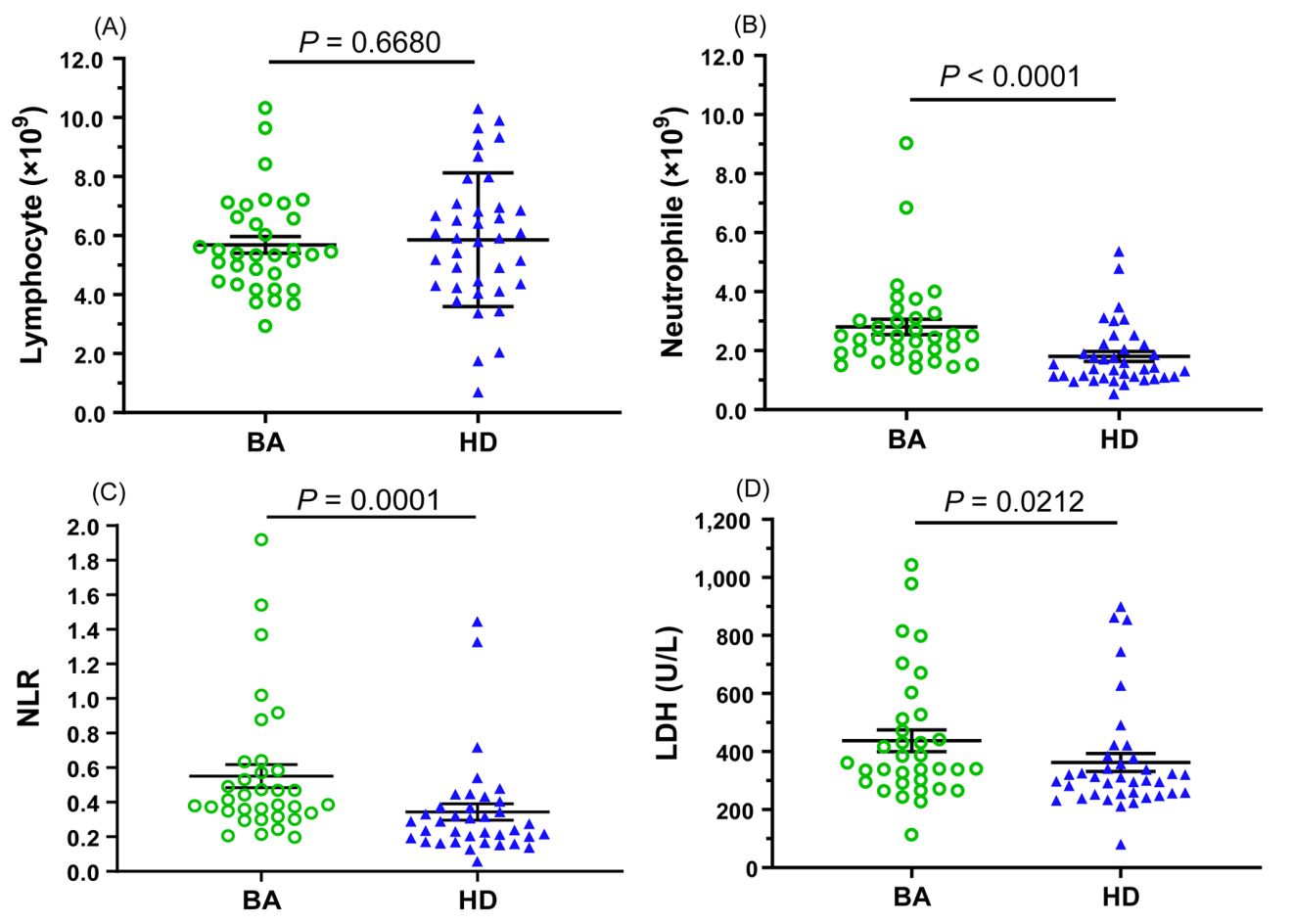


## Fig. S5 Comparison the levels of blood corpuscle, LDH between BA and HD

The levels of lymphocyte (A), neutrophile (B), NLR (C), and LDH (D) compared between BA and HD groups with expanded cases


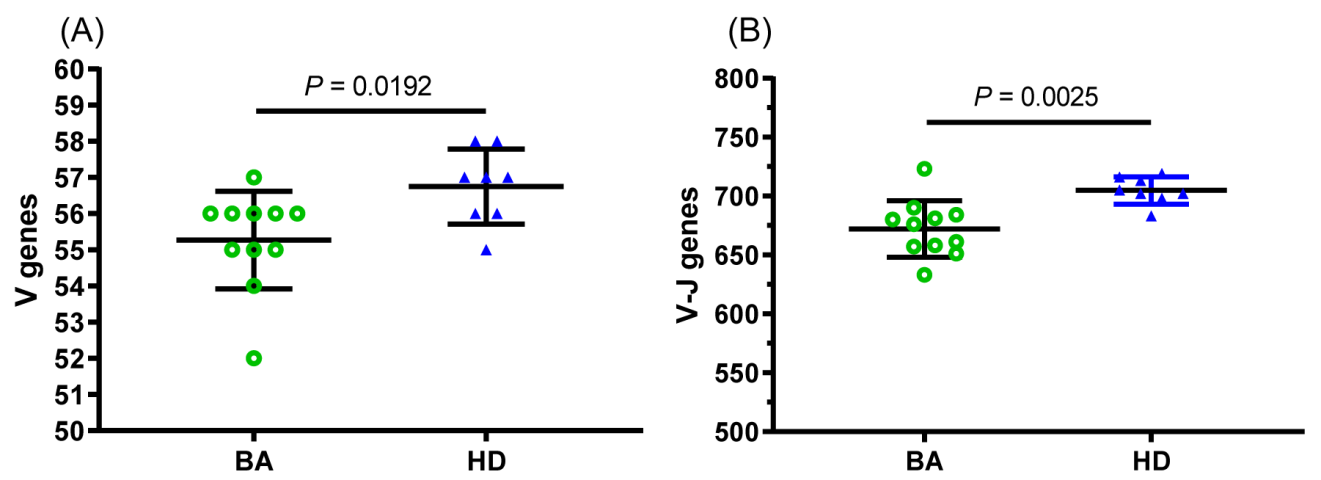


## Fig. S6 Usage of V, and VJ gene combinations in BA and HD samples

The number of different V gene segments (A), and VJ gene combinations (B) for each for BA and HD samples. The middle solid line is mean of counts.

Supplementary Table

Table S1 TRBV specificity groups from GLIPH2 analysis (see Supplementary file-2)

# Data availability

The datasets for this study can be found in the National Center for Biotechnology Information (NCBI) [Human sample from Homo sapiens - BioSample - NCBI (nih.gov)](https://www.ncbi.nlm.nih.gov/biosample/?LinkName=bioproject_biosample_all&from_uid=718110) . [PRJNA718110](https://www.ncbi.nlm.nih.gov/bioproject/718110) Homo sapiens.
